# Apoptosis induced in vivo by photodynamic therapy in normal brain and intracranial tumour tissue

**DOI:** 10.1054/bjoc.2000.1426

**Published:** 2000-10

**Authors:** L Lilge, M Portnoy, B C Wilson

**Affiliations:** Photonics Research Ontario, Department of Medical Biophysics and Ontario Cancer Institute, University of Toronto, 610 University Avenue, Toronto, Ontario, M5G 2M9

**Keywords:** photodynamic therapy, brain, apoptosis

## Abstract

The apoptotic response of normal brain and intracranial VX2 tumour following photodynamic therapy (PDT) mediated by 5 different photosensitizers (Photofrin, 5-aminolaevulinic acid (ALA)-induced protoporphyrin IX (PpIX), chloroaluminium phthalocyanine (AlCIPc), Tin Ethyl Etiopurpurin (SnET _2_), and *meta*-tetra(hydroxyphenyl)chlorin (*m* THPC)) was evaluated following a previous analysis which investigated the necrotic tissue response to PDT at 24 h post treatment. Free DNA ends, produced by internucleosomal DNA cleavage in apoptotic cells, were stained using a TUNEL (terminal deoxynucleotidyl transferase (TdT)-mediated dUTP nick-end labelling) assay. Confocal laser scanning microscopy (CLSM) was used to quantify the local incidence of apoptosis and determine its spatial distribution throughout the brain. The incidence of apoptosis was confirmed by histopathology, which demonstrated cell shrinkage, pyknosis and karyorrhexis. At 24 h post PDT, AlClPc did not cause any detectable apoptosis, while the other photosensitizers produced varying numbers of apoptotic cells near the region of coagulative necrosis. The apoptotic response did not appear to be related to photosensitizer dose. These results suggest that at this time point, a minimal and fairly localized apoptotic effect is produced in brain tissues, the extent of which depends largely on the particular photosensitizer. © 2000 Cancer Research Campaign


British Journal of Cancer (2000) 83(8), 1110–1117
© 2000 Cancer Research Campaign
doi: 10.1054/ bjoc.2000.1426, available online at http://www.idealibrary.com on IDEAL®

### Apoptosis induced in vivo by photodynamic therapy in normal brain and intracranial tumour tissue

**L Lilge[1,2], M Portnoy[3] and BC Wilson[1,2,3]**

[1]Photonics Research Ontario, [2]Department of Medical Biophysics and [3]Ontario Cancer Institute, University of Toronto, 610 University Avenue, Toronto, Ontario M5G 2M9

**Summary** The apoptotic response of normal brain and intracranial VX2 tumour following photodynamic therapy (PDT) mediated by 5 different photosensitizers (Photofrin, 5-aminolaevulinic acid (ALA)-induced protoporphyrin IX (PpIX), chloroaluminium phthalocyanine (AlClPc), Tin Ethyl Etiopurpurin ($SnET_2$), and *meta*-tetra(hydroxyphenyl)chlorin (*m*THPC)) was evaluated following a previous analysis which investigated the necrotic tissue response to PDT at 24 h post treatment. Free DNA ends, produced by internucleosomal DNA cleavage in apoptotic cells, were stained using a TUNEL (terminal deoxynucleotidyl transferase (TdT)-mediated dUTP nick-end labelling) assay. Confocal laser scanning microscopy (CLSM) was used to quantify the local incidence of apoptosis and determine its spatial distribution throughout the brain. The incidence of apoptosis was confirmed by histopathology, which demonstrated cell shrinkage, pyknosis and karyorrhexis. At 24 h post PDT, AlClPc did not cause any detectable apoptosis, while the other photosensitizers produced varying numbers of apoptotic cells near the region of coagulative necrosis. The apoptotic response did not appear to be related to photosensitizer dose. These results suggest that at this time point, a minimal and fairly localized apoptotic effect is produced in brain tissues, the extent of which depends largely on the particular photosensitizer. © 2000 Cancer Research Campaign

**Keywords**: photodynamic therapy; brain; apoptosis

An incidence of 15 000 new cases of primary confirmed brain tumours is reported in the US each year. The incidence rate increases with age up to 65, the most frequent type being glioblastoma multiforme (29%), meningioma (18%), anaplastic astrocytoma (11%) and astrocytoma (9%) and, in spite of maximal surgical resection and aggressive adjuvant radiotherapy and/or chemotherapy, the prognosis for these patients is poor, with a 1.8-year mean survival from the time of diagnosis (Mahaley et al, 1989).

Intraoperative treatment of malignant brain tumours by photodynamic therapy (PDT) has been investigated in several clinical trials (Muller and Wilson, 1991, 1996; Kostron et al, 1996). This technique involves administering a photosensitizer which, after a suitable time interval for uptake in the tumour, is activated by local application of light, resulting in the generation of cytotoxic photoproducts, notably singlet oxygen, $^1O_2$ (Dougherty and Marcus, 1992; Levy, 1994; Ochsner, 1997). The light is typically delivered either by interstitial optical fibres (Origitano and Reichman, 1993) or via an inflatable 'balloon' applicator which distributes the light uniformly throughout the resection cavity (Schmidt et al 1996; Muller and Wilson, 1996). These trials have shown evidence of improved survival, and multicentre Phase III prospectively randomized clinical trials of PDT in both de novo and recurrent astrocytic tumours using the haematoporphyrin derivative photosensitizer Photofrin (QLT Phototherapeutics Inc, Vancouver, BC, Canada) are currently in progress. However, some studies have also reported transient or permanent worsening of neurological function following PDT, with neurological impairment or neurocognitive deficit in a few patients (Muller and Wilson, 1996). Muller and Wilson (1992) also reported increased intracranial pressure post-operatively, attributed to PDT-induced oedema in the exposed normal brain. These clinical complications could limit the PDT dose and hence the anti-tumour efficacy achievable. This is particularly relevant to the use of new, second generation photosensitizers. While these may have higher tumour-to-normal brain selectivity, in addition to other advantages such as reduced skin photosensitization, greater light penetration at the longer wavelengths and higher $^1O_2$ yield, possible damage to normal brain tissue remains a critical concern.

In a previous pre-clinical study in a rabbit model bearing intracranially-implanted VX2 carcinoma (Lilge et al, 1996), we investigated necrosis of normal brain and tumour to PDT mediated by 4 different photosensitizers: Photofrin, ALA (5-aminolaevulinic acid: a precursor in haem biosynthesis which results in the photosensitizer protoporphyrin IX, PpIX), AlClPc (non-sulphonated chloroaluminium phthalocyanine) and $SnET_2$ (tin ethyl etiopurpurin). With one exception, all 4 drugs produced significant gross haemorrhagic necrosis in both tumour and normal brain tissue. The zones of necrosis were very sharply delineated, consistent with a 'threshold' model of PDT in which a minimum concentration of $^1O_2$ is required to produce necrosis (Chen et al, 1996; Lilge et al, 1996; Farrell et al, 1998). There was, however, considerable variation in the PDT threshold doses and in the tissue uptake between the photosensitizers. In general, the threshold values for normal brain were lower than those of tumour tissue, implying that normal brain is intrinsically more sensitive to PDT damage than

*Received 22 February 2000*
*Revised 6 June 2000*
*Accepted 7 June 2000*

*Correspondence to*: BC Wilson the tumour. Hence, clinical benefit depends on having higher drug concentration in tumour and accurate targeting of the treatment light. The exception was with ALA-PDT in white matter, where no necrotic damage was observed, probably due to lack of PpIX synthesis.

In addition to these gross necrotic effects on normal brain and brain adjacent to tumour (BAT), Yoshida et al (1992a) have reported histopathologic evidence of lethal injury to neurons, extending beyond the zone of coagulative necrosis and developing over a period of 18 h after Photofrin-PDT in a rat model. If generally true, this would increase further the concern over PDT damage to normal brain structures. Hence, the focus of the present study was to investigate sub-necrotic cell damage in brain, in particular the occurrence of apoptosis induced in vivo.

Apoptosis has not yet been demonstrated in the PDT response of intracranial tissues. However, PDT-induced apoptosis has been shown in vitro in carcinoma cell lines (He et al, 1994; Noodt et al, 1996) and in other tissues in vivo, as an early event occurring within the first 24 hours after treatment (Zaidi et al, 1993). Also, following ionizing radiation therapy of the central nervous system, a significant proportion of patients develop late complications, including demyelination of nerve fibres and white matter necrosis (Wong et al, 1994; Schultheiss et al, 1995; Laperriere et al, 1997), and animal model studies (Li et al, 1996a,b) have confirmed radiation-induced apoptosis in normal brain tissues. Hence, apoptosis is certainly a possible mechanism of cell death following injury to normal brain. While PDT-induced necrosis usually results from direct photochemical injury to the cells or from destruction of tissue vasculature with subsequent ischaemic hypoxia, apoptosis is considered a deliberate response to specific, but often indirect stimuli (Majno and Joris, 1995; White, 1996). The response characteristics may, therefore, be very different.

The present study represents an initial investigation of PDT-induced apoptosis in normal brain and intracranial tumour. It is based on applying, retrospectively, an assay for apoptosis in brain tissues banked from our previous studies of PDT-induced necrosis in rabbit brain/tumour (Lilge and Wilson, 1998). While this limited the range of treatment parameters that could be investigated, and in particular provided data only at 24 h post treatment, it served to determine whether or not PDT-induced apoptosis does occur in brain tissues and if it is photosensitizer dependent, and to guide future systematic prospective studies. *m*THPC (*meta*-tetrahydroxyphenyl chlorin) has since been added to our pre-clinical necrosis studies with Photofrin, ALA, AlClPc and $SnET_2$, and so is also included here.

### MATERIALS AND METHODS

### Animal model and PDT treatments

The animal model and PDT procedures have been described in detail previously (Lilge and Wilson, 1998). Briefly, rabbits (New Zealand Whites, male, 2–3.5 kg) were used since the brain is large enough to grow tumours 8–12 mm in diameter without acute morbidity, which facilitates determination of the PDT threshold dose. No glial tumour is available for the rabbit, so a VX2 carcinoma was chosen. This exhibits some relevant characteristics of primary brain tumours, such as microinvasion, pseudo-palisading, and growth along blood vessels and in perivascular spaces, and causes breakdown of the blood–brain barrier (BBB) within the tumour and in brain adjacent to tumour (BAT).

Animals were randomized for tumour implantation, photosensitizer type and dose, and time interval before irradiation. Controls were either drug/no light or light/no drug. From the set of post-treatment brain sections obtained in our previous necrosis study (Lilge and Wilson, 1998), a subset was selected for evaluation of apoptosis, as listed in Table 1, which also includes the additional animals treated using *m*THPC. PDT was performed on the left hemisphere (see below), and the contralateral hemisphere served as a control in each animal to test for apoptosis due to photosensitizer alone or for secondary whole-brain effects (e.g. due to raised intracranial pressure). For tumour implantation, a burr hole was drilled 5 mm posterior to the sagittal suture and 5 mm left of the midline, through which $10^5$ VX2 cells in 50 μl phosphate buffered saline (PBS) were injected 6 mm below the dura. A 13-day growth period yielded 8–12 mm diameter tumours.

Photofrin (profimer sodium) was supplied as a freeze-dried powder (QLT Phototherapeutics, Vancouver, BC, Canada) and reconstituted to 5 mg/ml in 5% dextrose. ALA in hydrochloride form (Sigma Scientific, St. Louis, MO, USA) was dissolved in

**Table 1** Summary of PDT treatment groups

| Treatment group | Photosensitizer | Light delivered energy, wavelength (J, nm) | Injected dose (mg $kg^{-1}$) | Drug-light time interval (h) | No. of animals tumour-bearing | No. of animals normal |
|---|---|---|---|---|---|---|
| 1 | Photofrin | 50,514 | 2.5 | 24 | 3 | |
| 2 | Photofrin | 50,514 | 10 | 24 | 3 | 1 |
| 3 | ALA/PpIX | 50,514 | 20 | 6 | 3 | 1 |
| 4 | ALA/PpIX | 50,514 | 100 | 6 | 3 | 1 |
| 5 | AlClPc | 50,514 | 0.2–0.5 | 24 | 4 | 1 |
| 6 | *m*THPC | 50,514 | 0.3 | 24 | | 3 |
| 7 | $SnET_2$(liposome) | 50,514 | 1.0 | 24 | 2 | |
| 8 | $SnET_2$ (emulsion) | 50,51A,660 | 1.0 | 24 | 2 | 1 |
| Control groups | | | | | | |
| I | Photofrin | 0,- | 2.5 | | 2 | |
| II | AlClPc | 0,- | 0.2 | n/a | | 1 |
| III | None | 50,514 | 0 | | | 1 |

© 2000 Cancer Research Campaign

PBS (80 g/ml) and the pH was adjusted to 6.5–6.8 with 1N NaOH: this value was selected rather than neutral or slightly basic pH in order to keep the injectate volume as low as possible. AlClPc was supplied in an emulsion carrier (1 mg/ml) by Dr Nancy Oleinick (Case Western Reserve University, Cleveland, OH, USA). $SnET_2$ (Purlytin; Miravant, Santa Barbara, CA, USA) was supplied ready for injection in 2 different delivery vehicles: emulsion ($SnET_2$-e) at 1 mg/ml and liposomes ($SnET_2$-l) at 0.87 mg/ml. *m*THPC (Scotia Drug Discovery, London, UK) was reconstituted to 0.5 mg/ml in 30% polyethylene glycol, 20% ethanol and 50% distilled water (Scotia Pharma Ltd, London, UK). All photosensitizers were injected intravenously at <1 ml/min, either 24 h or (for ALA) 6 h before light irradiation.

For light application, a left parietal craniotomy (12 × 8 mm) was performed and the dura removed. Light (50 J at 35 mW) from an argon laser (Ion-Laser Technologies, Salt Lake City, UT, USA) was delivered via a cleaved-end optical fibre to a depth of 6 mm into the centre of the tumour or at the same anatomical site in non-tumour bearing animals. A wavelength of 514 nm was used to limit the light penetration and hence the size of the PDT-induced lesions, while achieving adequate drug activation. Two animals given $SnET_2$-e were irradiated at the absorption maximum of the drug (660 nm) in order to assess the effect of the larger treatment volume. Following treatment, the animals were allowed to recover and analgesic (0.2 ml Temgesic, Reckitt-Colman, Hull, UK) was then administered subcutaneously every 8 h. The animals were sacrificed 24 h or (for $SnET_2$) 72 h post treatment by lethal injection (T-61: Hoechst, Regina, SK, Canada), by which time necrosis was fully developed (Yoshida et al, 1992b; Lilge and Wilson, 1998).

The brains were removed intact, fixed in 10% formalin in PBS for 10 days, and cut into 3 mm thick transverse slices. The slice displaying the largest diameter of necrosis was processed and embedded in paraffin and serial 4–6 µm sections were cut. The TUNEL assay (Promega, Madison, WI, USA) for apoptosis was applied to these sections, comprising terminal deoxynucleotidyl transferase (TdT)-mediated dUTP nick-end labelling, and staining with fluorescein-12-dUTP® to identify exposed 3′ ends in the DNA of apoptotic cells by the green fluorescein fluorescence. Sections were counter-stained with propidium iodide (1 µg/ml in PBS–$CaCl_2$, $MgCl_2$: Molecular Probes, Eugene, OR, USA), which stained all nucleated cells with red fluorescence. Finally, the sections were treated with SlowFade™-Light Antifade (Molecular Probes, Eugene, OR, USA) cover medium. Positive controls were made during each staining session by applying 100 µl of DNase I (GibcoBRL, Burlington, ON, Canada) to a randomly-selected section prior to treatment with TdT, resulting in chromosomal fragmentation and exposure of multiple 3′ ends of DNA. Negative controls were created by incubation of sections with a buffer containing fluorescein-12-dUTP®, but no TdT. Randomly selected sections representing all treatment groups were stained during each session to minimize the impact of uncontrolled variations in staining.

A fluorescence confocal laser-scanning microscope (CLSM: BioRad MRC 600, Mississauga, ON, Canada) was used to map the spatial distribution and density of apoptotic bodies. These were identified using shareware imaging software (Confocal Assistant Version 4.02) to generate false-colour images, encoding propidium iodide and fluorescein as red and green, respectively. The fluorescence excitation wavelength for propidium iodide was 564 nm, with detection at >600 nm: fluorescein was excited at 488 nm with detection at 535 ± 25 nm. Red blood cells (RBC) showed false-positive green fluorescein staining which could not be distinguished automatically from apoptotic cells. Hence, sections were scanned by eye to identify the latter on the basis of size and morphology (condensed chromatin within the cytoplasm and lack of biconcave shape). Using 600–1000 × magnification, 2 or 3 complete cross-sections from each brain were scanned by moving the microscope stage in X and Y in 1 mm increments. The apoptotic bodies were counted in each field-of-view, yielding a 2D distribution of the apoptotic cells. The actual position of the treatment fibre tip (centre of the light field) could not be visualized in the sections. Hence, the geometric centre of the measured apoptotic cell distribution was determined from the X and Y distributions, assuming that the geometric centre represents the position of the treatment fibre tip. The radial distribution of apoptotic cell density, D(r), (no. bodies per $mm^2$) was thereby calculated for each section. These were then averaged over all sections for each animal and then for all animals in each treatment group. From light diffusion theory, the light fluence dependence on radial distance from a point source is of the form $H(r) \propto e^{-\mu eff/r}$, where $\mu_{eff}$ is the effective optical attenuation coefficient of the tissue at the treatment wavelength. Hence, plots of ln{r.D(r)} versus r were compared with the corresponding radial light fluence distribution, as measured previously (Lilge and Wilson, 1998).

In order to confirm, qualitatively, the occurrence and distribution of apoptosis, selected tissue sections directly adjacent to those used for the TUNEL assay were H&E stained and examined under a light microscope for cell shrinkage, karryorhexis, and pyknosis, all of which are associated with apoptotic cell response (Darzynkiewics et al, 1994).

### RESULTS

Figure 1 shows typical examples of CLSM images with the TUNEL assay, demonstrating positive fluorescein against the background of propidium iodide-stained cells. Light microscopy of H&E-stained sections exemplifies, as in Figures 2 and 3, the apoptotic characteristics of cell shrinkage, chromosomal fragmentation, and darkly-staining chromatin abutting nuclear envelopes.

The validity of the TUNEL assay applied to these brain sections was tested using the positive and negative control slides. Approximately 70% of the DNase-treated cells in the former demonstrated green fluorescence, verifying the TdT labelling of the exposed 3′ ends of DNA. None of the negative controls showed green fluorescence, indicating that the incorporation of Fluorescein-12-dUTP® was mediated by TdT. There was also good correspondence between the H&E and TUNEL identification of apoptotic cells for all treatment groups, although the former was not quantified.

The PDT-treatment control groups (I–III) with photosensitizer or light alone showed minimal apoptosis, with an average of 3.7 apoptotic cells for the whole brain sections. The contralateral hemispheres in all animals in treatment groups 1–7 also demonstrated only 2–3 apoptotic cells on average, and this was supported by the corresponding H&E-stained sections, that showed neither necrosis nor cells with apoptotic features. Hence, there was no evidence of a 'global' brain response in this model with any photosensitizer when the treatment volume was limited by the use of short wavelength (514 nm) light. However, in group 8 treated with $SnET_2$-e and

*© 2000 Cancer Research Campaign*

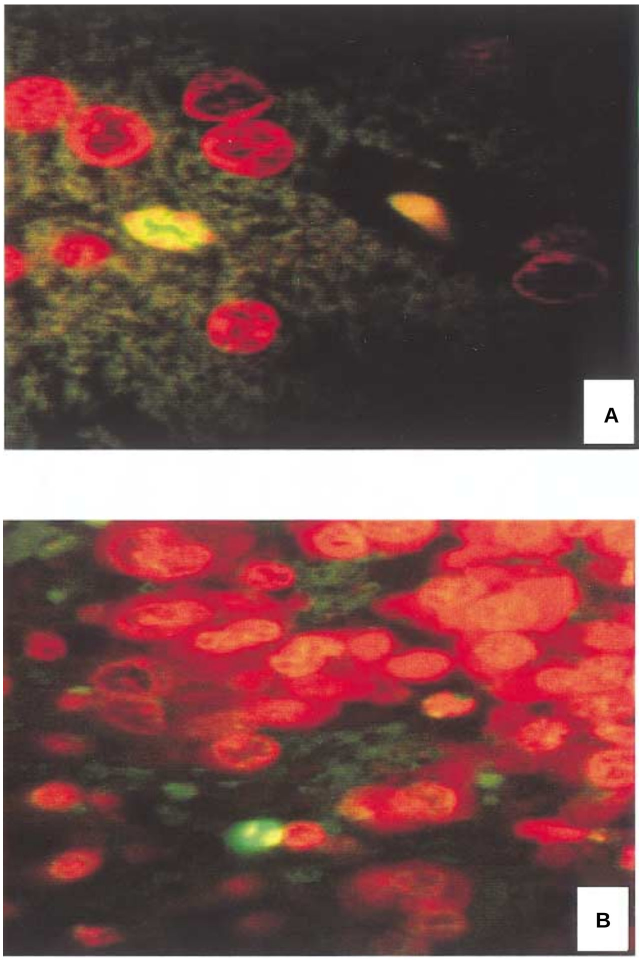

**Figure 1** Confocal fluorescence images of fluorescein-stained apoptotic cells (bright green) in brain sections of tumour-bearing animals (1000×). The red cells are propidium iodide-stained. (**A**) 1 mg/kg $SnET_2$-e, 50 J at 660 nm. (**B**) 2.5 mg/kg Photofrin, 50 J at 514 nm

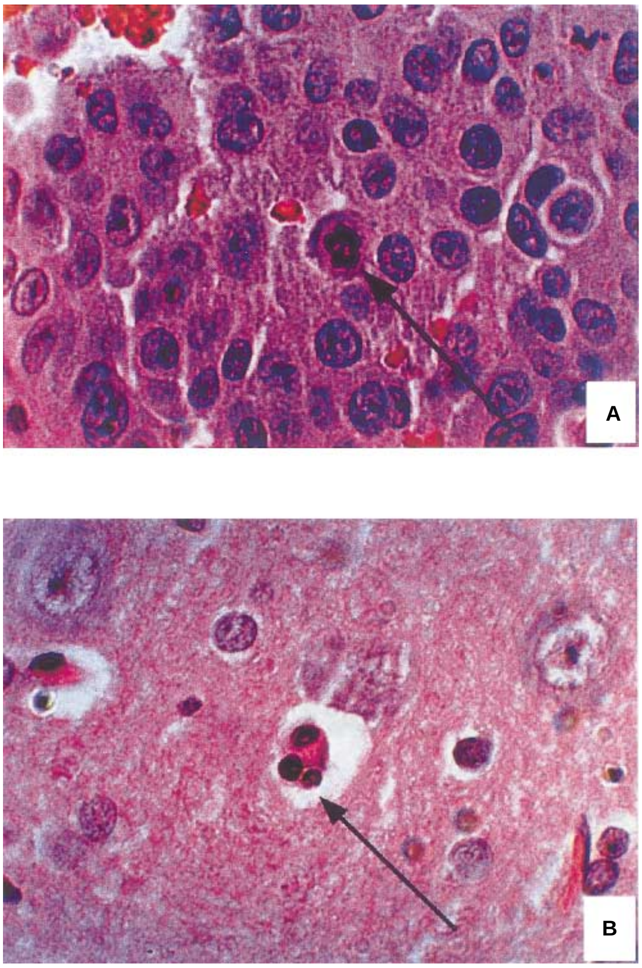

**Figure 2** H&E-stained micrographs of tumour-bearing brain sections treated with 50 J at 514 nm (1000×). (**A**) 20 mg/kg ALA, sacrificed 24 h post PDT. (**B**) 1 mg/kg $SnET_2$-l, sacrificed 72 h post PDT. Both sections exhibit condensed chromatin packed in smooth masses against the cell membrane (arrows)

**Table 2** Total number of apoptotic cells in the treated hemispheres of individual animals, and treatment group averages

| **Group**<br>**photosensitizer**<br>**PS dose**<br>**(mg kg$^{-1}$)** | **1**<br>**Photofrin**<br>**2.5** | **2**<br>**Photofrin**<br>**10** | **3**<br>**ALA-PpIX**<br>**20** | **4**<br>**ALA-PpIX**<br>**100** | **5**<br>**AlClPc**<br>**0.5** | **6**<br>**mTHPC**<br>**5** | **7**<br>**$SnET_2$-l**<br>**0.25–1.0** | **8**<br>**$SnET_2$-e**<br>**1.0** |
|---|---|---|---|---|---|---|---|---|
| | 93 | 59 | 3 | 17 | 0 | 20[a] | 29 | 1 |
| | 41 | 4 | 15 | 17 | 0 | 17[a] | 2 | 95[b] |
| | 39 | 83 | 14 | 6 | 1 | 12[a] | | 263[b] |
| | 34[a] | 61[a] | 49[a] | 5[a] | 4[a] | 10[a] | | |
| Average | 52 ± 28 | 52 ± 34 | 20 ± 20 | 11 ± 7 | 2 ± 2 | 15 ± 5 | 16 + 19/– 16 | 179[h] |
| Drug dose average | 52 ± 29 | | 16 ± 15 | | | | | |

[a] Non-tumour bearing animal; [b]treated at 660 nm

660 nm light, there was extensive necrosis and apoptosis, including the contralateral hemisphere. This is most likely due to the larger effective treatment volume.

Table 2 shows the total number of apoptotic cells observed in the treated hemispheres for each animal, together with the treatment-group averages. Note that there is considerable animal-to-animal variability in these data. In part, this appears to be due to the presence of ‘outliers’. For example, in Group 2 one animal had much lower counts than the other 7 Photofrin-PDT animals. Similarly, in Group 8 the animal treated at 514 nm had few apoptotic bodies compared to the 2 animals treated at 660 nm, although in this case the difference could reflect a true biological effect.

*© 2000 Cancer Research Campaign*

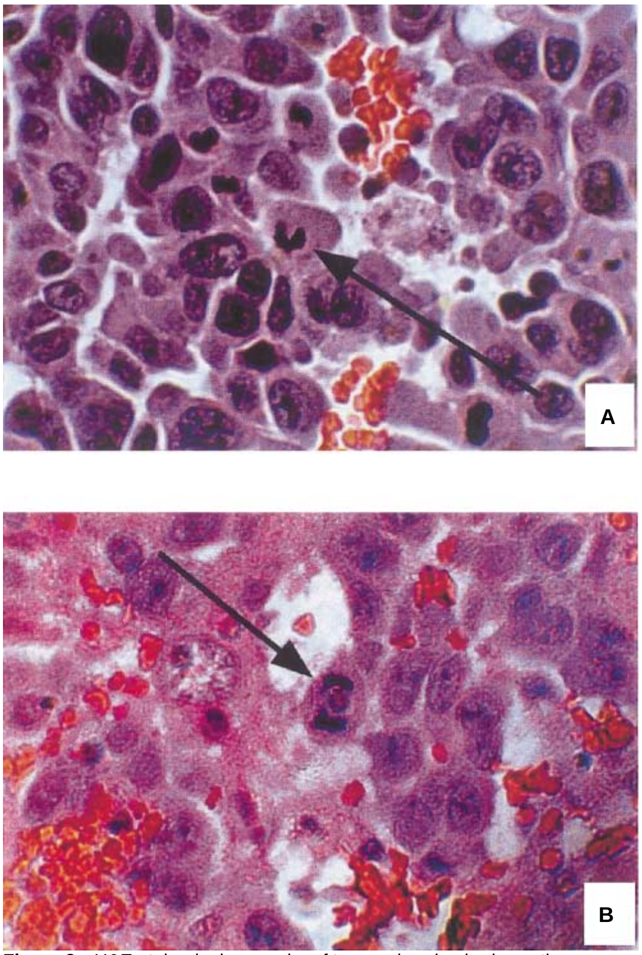

**Figure 3** H&E-stained micrographs of tumour-bearing brain sections treated with 50 J at 514 nm (1000×). (**A**) 10 mg/kg Photofrin, sacrificed 24 h post PDT. (**B**) 1 mg/kg $SnET_2$-l, sacrificed 72 h post PDT. Both sections exhibit sickle-shaped pyknotic chromatin packed in smooth masses against the nuclear envelope (arrows). Eosin-stained red blood cells are present within the region of coagulative necrosis

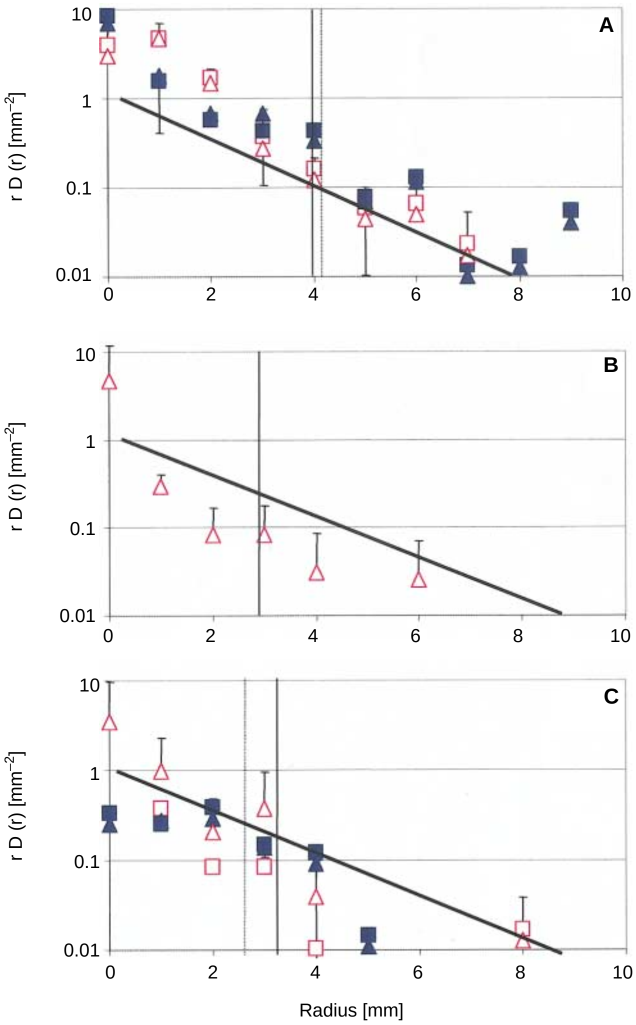

**Figure 4** Semilog plot of r times apoptotic cell density ($mm^{-2}$) *vs* radius (mm) for (**A**) Photofrin; (**B**) ALA-PpIX; (**C**) *m*THPC. Open symbols: low drug dose. Solid symbols: high drug dose (Table 1). Squares: tumour-bearing animals. Triangles: averages for all animals treated using the same parameters. Vertical lines: radius of necrosis for low (dashed) and high (solid) drug doses (Lilge and Wilson, 1998). Bold sloped line: light fluence rate (normalized to power delivered at the fibre tip) (Lilge et al., 1996)

Photofrin-PDT produced the highest average count of 52 ± 29 per whole hemisphere section. ALA-PpIX, *m*THPC, and $SnET_2$-1 produced intermediate levels of 16 ± 15, 15 ± 5, and 16 + 19/–16, respectively, while AlClPc gave counts comparable to controls. Direct comparisons of these values should be made with caution, however, since they have not been corrected for differences in drug concentration, which vary by at least an order of magnitude between photosensitizers (Lilge and Wilson, 1998), nor for their different optical extinction coefficients.

In most brain sections, the apoptotic cells were localized primarily near the region of PDT-induced necrosis or in small pockets of viable cells within the necrotic zone. Figure 4 shows the radial distributions of apoptotic cells for treatment groups 1–4 and 6, averaged over all animals treated using the same parameters. The corresponding graphs for treatment groups 5 and 7 are not shown due to the low counts, while group 8 is not shown since the distribution was very diffuse. The radius of PDT-induced necrosis in normal brain, as measured previously in these treatment groups (Lilge and Wilson, 1998) is also indicated on these graphs: 3.7 ± 0.4, 3.8 ± 0.4, >2.5, 3.1, 2.8 ± 0.4 mm for groups 2–6, respectively. It can be seen that apoptosis occurs even beyond the (sharp) boundary of frank necrosis in all these groups.

Figure 4 also shows the previously-determined radial distribution of light fluence in the tissue. In the case of Photofrin and *m*THPC (Figures 4A,C) the slopes of the apoptotic cell density distribution are comparable to that of the light fluence, with ln{r.D(r)} having an approximately linear decrease with r. Regression fits of the radial density distributions yielded equivalent effective attenuation coefficients of 0.72–0.78 $mm^{-1}$, comparable to the value of 0.8 ± 0.3 $mm^{-1}$ measured directly (Lilge et al, 1996). The correlation coefficients were highest for Photofrin-PDT ($R^2 > 0.85$). Although the correlation was weakest for ALA-PDT ($0.87 > R^2 > 0.69$), there was still a clear decrease in apoptosis with distance from the light source (Figure 4B). No evidence of apoptotic cells was noted within the white matter for ALA-PDT, while apoptotic cells were observed in this compartment for animals treated with Photofrin, *m*THPC and $SnET_2$-l.

*© 2000 Cancer Research Campaign*

### DISCUSSION

Apoptosis is not a synchronous process (Majno and Joris, 1995), so that cells at different stages of apoptotic death will coexist in the same tissue section. The in situ assay employed here can detect only the intermediate stages – DNA fragmentation, chromatin condensation and formation of apoptotic bodies – but not the early or late stages, such as loss of membrane asymmetry and phagocytosis. Hence, our measurements will underestimate the total number of cells that undergo apoptosis in response to PDT damage. Furthermore, the use of the previously-banked tissues from animals sacrificed at a late time point after treatment (to allow full development of necrosis) has not provided information on the time course of apoptosis, nor identification of the cell types involved. Clearly, these are important issue in terms of assessing the true significance of PDT-induced apoptosis in the brain. Although we have not distinguished between tumour and normal cells scoring apoptosis, there were no statistically-significant differences between the total counts in tumour-bearing and non-tumour bearing animals at the 24 h time point. This is consistent with previous findings for necrosis (Lilge and Wilson, 1998) where, with the exception of the absence of necrosis in white matter with ALA-PDT, the threshold dose values for a given photosensitizer were of the same order of magnitude for both tumour and normal brain tissue. It is noteworthy that apoptosis also was not seen in white matter for ALA-PDT.

The control animals treated with light or photosensitizer alone, and the contralateral hemispheres in PDT-treated animals where the volume of PDT-induced necrosis was restricted (groups 1–7), showed background levels of apoptosis. Thus, it is reasonably certain that the apoptosis seen in the treated tissue is attributable to the local photodynamic effect, whether direct cell killing or secondary local microvascular damage.

The total apopotic counts and the volumes of PDT-induced necrosis (Lilge and Wilson, 1998) show the same qualitative trends in some cases: for example, the volumes of necrosis corresponding to the data in Table 2 were ~195, 125 and 22 $mm^3$ for Photofrin, ALA (tumour or grey matter) and $SnET_2$-1, respectively. By contrast, AlClPc does not appear to produce any significant level of apoptosis at 24 h post treatment, even although significant necrosis was seen (~65 $mm^3$). Chan et al (1996) and Margaron et al (1996) have shown that phthalocyanines localize differently in cells in vitro than either Photofrin or ALA-PpIX. Since $^1O_2$, which is generated by all these photosensitizers, is very short-lived and thus acts within <~0.1 µm of its site of generation (Tyrrell, 1999), the intracellular sites of PDT damage will be different for AlClPc and so might not activate the apoptotic pathways. This lack of consistent correlation between necrosis and apoptosis suggests that the latter is not simply a secondary response to the presence of necrotic tissue.

The apoptotic counts were also not dependent on the drug dose for any of the photosensitizers, at least within the range of doses used here, suggesting that apoptosis does not occur exclusively from direct PDT cellular damage, but is at least partly a consequence of secondary effects, such as microvascular damage, brain swelling or hypoxia. The presence of apoptotic cells at this relatively long time after treatment also supports this interpretation, since PDT-induced apoptosis in vitro is typically complete within a few hours (Oleinick and Evans, 1998; Separovic et al, 1998). It is known that there is a large vascular component in the necrotic response of normal brain tissue (Dereski et al, 1991) and intracranial 9L glioma (Chopp et al, 1996) to Photofrin-PDT, whereas in other tumour models aluminium phthalocyanine operates predominantly by direct cell kill (Peng et al, 1990). This is consistent with the much larger apoptotic counts seen here with Photofrin than with phthalocyanine, if secondary effects are indeed important in the apoptotic response of brain tissue.

The rabbits treated with $SnET_2$-e at 660 nm demonstrated the largest apoptotic response of all treatment groups. However, this was not localized to the region of necrosis or the treated hemisphere. On the basis of the optical penetration depth in brain tissue (Eggert and Blazek, 1993; Lilge and Wilson, 1998), the effective treatment volume is ~8-fold larger at 660 nm than at 514 nm (but still confined to the treatment hemisphere). This could cause increased intracranial pressure and, hence, apoptosis may be a result of secondary damage.

The form of the spatial distribution of apoptotic cells in the tissue was quite variable (sometimes centrally-peaked and in other cases more torroidal in shape), so that the method of determining the geometric centre of the distribution was necessarily somewhat arbitrary and may not coincide exactly with the location of the treatment light source. Nevertheless, the strong correlation for some photosensitizers between the fluence distribution and the radial apoptotic cell density (Figure 4) suggests that the measurements are not markedly skewed. These data also demonstrate that, for these treatment parameters and time post treatment, the local rate of PDT-induced apoptosis is proportional to the local light fluence (and/or fluence rate, since the delivered light power was not varied here). This is a necessary, but not sufficient, condition for PDT-induced apoptosis also to have a threshold behaviour. The threshold doses would then be lower than those for necrosis, since apoptosis is seen beyond the necrotic boundary. We have recently confirmed this quantitatively for Photofrin-PDT (Lilge et al, 2000), the threshold doses for apoptosis and necrosis in normal rat brain being estimated as $0.6–2.7.10^{17}$ photons $cm^{-3}$ and $2.2.10^{18}$ photons $cm^{-3}$, respectively (minimum number of photons absorbed by the photosensitizer per unit tissue volume to produce the specific biological endpoint).

The predominant brain lesion seen following PDT with 514 nm light is coagulative necrosis, since there are relatively few apoptotic cells beyond the necrotic boundary: e.g. for Photofrin-PDT ~8,000 apoptotic cells would be present outside the necrotic boundary according to the measurements from groups 1 and 2. This might be interpreted to mean that apoptosis is not a major contributor to damage to BAT and, therefore, will not be clinically significant. However, the effect may be much greater at earlier times. By analogy, in the case of ionizing radiation, Li et al. (1996b) reported apoptosis in rat spinal cord peaking at 8 h, returning to baseline levels after 24 h and, based on in vitro results (He et al, 1994; Noodt et al, 1996) apoptosis due to direct cellular effects of PDT is likely to occur on an even shorter time scale.

### CONCLUSIONS

This study demonstrates that intracranial PDT in vivo can produce measurable apoptosis 24 h post treatment. Furthermore, the apoptosis can extend beyond the boundary of frank necrosis, while the number of apoptotic cells depends on the photosensitizer used. However, it does not appear to depend on the photosensitizer dose, within the ranges used. At the same time, for Photofrin, *m*THPC and, to a lesser extent, ALA-PpIX, the apoptotic cell density and light fluence distributions correlate, demonstrating a strong local

© 2000 Cancer Research Campaign 

PDT 'dose' dependence. Reconciling these somewhat contradictory observations will require detailed examination of the roles of direct cell killing versus indirect, e.g. vascular, effects in brain tissue.

The extension of apoptosis beyond the boundary of necrosis has potentially important clinical implications, since it may increase the extent of normal brain damage beyond the intended treatment depth, with consequent increase in neurological risk. Conversely, since apoptosis is not accompanied by the inflammatory response characteristic of necrosis (White, 1996), selective PDT induction of localized apoptosis in brain tumours could be preferable to a necrotic response. Hence, there could also be an opportunity for improved clinical results if PDT-induced apoptosis can be exploited.

A critical issue for future pre-clinical studies will be to determine the time course of PDT-induced apoptosis post treatment for the different photosensitizers and treatment conditions, in order to assess the maximum level of apoptosis that occurs beyond the necrotic zone. This work is in progress. It is also important for optimizing PDT dosimetry and clarifying the mechanisms of action to confirm that apoptosis does follow a threshold model of PDT response. A systematic drug dose, light dose and drug-light time interval study with each photosensitizer is required in order to quantify the relative contributions of apoptosis and necrosis to the tissue response for different treatment parameters. For example, recent preliminary data (Lilge et al, 2000) show much more apoptosis in normal brain with Photofrin-PDT at very low light doses (1 J $cm^{-2}$) that do not cause necrosis than with doses (17 J $cm^{-2}$) which do induce necrosis. Hence, it is not possible simply to extrapolate data from high to low doses, since apoptosis and necrosis are competing, or possibly even antagonistic, responses.

### ACKNOWLEDGEMENTS

This work was supported by the National Cancer Institute of Canada. The authors wish to thank Dr YQ Li for his help interpreting the H&E slides, P Pournazari, A Lee, J Park, and M Sepers for assistance with the in vivo experiments, R DaCosta for help with confocal microscopy and Drs Muller and S Wong for helpful discussions. Photofrin was kindly provided by QLT Phototherapeutics Inc, Vancouver, BC, Canada, $SnET_2$ (Purlytin) by Miravant Inc, Santa Barbara, CA, USA, *m*THPC by Scotia Drug Discovery, London, UK and AlClPc by Dr Nancy Oleinick, Case Western Reserve University, Cleveland, OH, USA.

### REFERENCES

Chan WS, Brasseur N, La Madeleine C and Van Lier JE (1996) Evidence for different mechanisms of EMT-6 tumor necrosis by photodynamic therapy with disulphonated aluminum phthalocyanine or Photofrin: Tumor cell survival and blood flow. *Anticancer Res* **16**: 1887–1892

Chen Q, Chopp M, Madigan L, Dereski MO and Hetzel FW (1996) Damage threshold of normal rat brain in photodynamic therapy. *Photochem Photobiol* **64**: 163–167

Chopp M, Dereski MO, Madigan L, Jiang F and Logie B (1996) Sensitivity of 9L gliosarcomas to photodynamic therapy. *Rad Res* **146**: 461–465

Darzynkiewics Z, Li X, and Gong J (1994) Assays of cell viability: Discrimination of cells dying by apoptosis. *Methods in Cell Biology* **41**: 15–38

Dereski MO, Chopp M, Garcia JH and Hetzel FW (1991) Depth measurements and histopathological characterization of photodynamic therapy generated normal brain necrosis as a function of incident optical energy dose. *Photochem Photobiol* **54**: 109–112

Dougherty TJ and Marcus SL (1992) Photodynamic therapy. *Eur J Cancer* **28A**: 1734–1742

Eggert HR and Blazek V (1993) Optical properties of normal human intracranial tissues in the spectral range of 400 to 2500 nm. *Adv Exp Med Biol* **333**: 47–55

Farrell TJ, Wilson BC, Patterson MS and Olivo MC (1998) Comparison of the in vivo photodynamic threshold dose for Photofrin, mono- and tetrasulfonated aluminum phthalocyanine using a rat liver model. *Photochem Photobiol* **68**: 394–399

Gal D (1994) Hunt for singlet oxygen under in vivo conditions. *Biochem Biophys Res Com* **202**: 10–16

He XY, Sikes RA, Thomsen S, Chung LWK and Jacques SL (1994) Photodynamic therapy with Photofrin II induces programmed cell death in carcinoma cell lines. *Photochem Photobiol* **59**: 468–473

Kostron H, Obwegeser A and Jakober R (1996) Photodynamic therapy in neurosurgery: A review. *J Photochem Photobiol* **B36**: 157–168

Laperriere NJ, Cerezo L, Milosevic MF, Wong CS, Patterson B and Panzarella T (1997) Primary lymphoma of brain: Results of management of a modern cohort with radiation therapy. *Radiother Oncol* **43**: 247–252

Levy JG (1994) Photosensitizers in photodynamic therapy. *Sem Oncol* **21**: 4–10

Li YQ, Jay V and Wong CS (1996a) Oligodendrocytes in the adult rat spinal cord undergo radiation-induced apoptosis. *Cancer Res* **56**: 5417–5422

Li YQ, Guo YP, Jay V, Stewart PA and Wong CS (1996b) Time course of radiation-induced apoptosis in the adult rat spinal cord. *Radiother Oncol* **39**: 35–42

Lilge L and Wilson BC (1998) Photodynamic therapy of intracranial tissues: A preclinical comparative study of four different photosensitizers. *J Clin Laser Med Surg* **16**: 81–91

Lilge L, Olivo MC, Schatz SW, Maguire SA, Patterson MS and Wilson BC (1996) The sensitivity of normal brain and intracranially implanted VX2 tumour to interstitial photodynamic therapy. *Br J Cancer* **73**: 332–343

Lilge L, Ching E, Portnoy M, Molckovsky A and Wilson BC (2000) Photofrin mediated PDT in normal rat brain: Assessment of apoptosis as a quantitative biological endpoint. *Proc SPIE* **3909**: 45–52

Mahaley MS, Mettlin C, Matarajan N, Law ER and Peace B (1989) National survey of patterns of care for brain-tumour patients. *J Neurosurg* **71**: 826–836

Majno G and Joris I (1995) Apoptosis, oncosis and necrosis: An overview of cell death. *Am J Path* **146**: 3–15

Margaron P, Madarnas P, Quellet R and Van Lier JE (1996) Biological activities of phthalocyanines. XVII Histopathologic evidence for different mechanisms of EMT-6 tumor necrosis induced by photodynamic therapy with disulfonated aluminum phthalocyanine or Photofrin. *Anticancer Res* **16**: 613–620

Muller PJ and Wilson BC (1992) Photodynamic therapy for brain tumors. In *A Clinical Manual: Photodynamic Therapy of Malignancies*, McCaughan JS (ed.) pp 201–211. RG Landes Co: Boca Raton, FL

Muller P and Wilson B (1991). Photodynamic therapy of brain tumors: Post-operative 'field fractionation'. *J Photochem Photobiol* **9B**: 117–119

Muller PJ and Wilson BC (1996) Photodynamic therapy for malignant newly diagnosed supratentorial gliomas. *J Clin Laser Med Surg* **14**: 263–270

Noodt BB, Berg K, Stokke T, Peng Q and Nesland JM (1996) Apoptosis and necrosis induced with light and 5-aminolaevulinic acid-derived protoporphyrin IX. *Br J Cancer* **74**: 22–29

Ochsner M (1997) Photophysical and photobiological processes in the photodynamic therapy of tumours. *J Photochem Photobiol* **B39**: 1–18

Oleinick NL and Evans HH (1998) The photobiology of photodynamic therapy: Cellular targets and mechanisms. *Rad Res* **150**: 146–156

Origitano TC and Reichman OH (1993) Photodynamic therapy for intracranial neoplasms: development of an image-based computer-assisted protocol for photodynamic therapy of intracranial neoplasms. *Neurosurg* **32**: 587–95

Peng QM, Nesland JM and Remington C (1990) Aluminum pthalocyanines with asymmetrical lower sulfonation and with symmetrical higher sulfonation: A comparison of localizing and photosensitizing mechanisms in human tumor LOX xenografts. *Int J Cancer* **46**: 719–726

Popovic EA, Kaye AH and Hill JS (1996). Photodynamic therapy of brain tumors. *J Clin Laser Med Surg* **14**: 251–261

Separovic D, Mann KJ and Oleinick NL (1998) Association of ceramide accumulation with photodynamic treatment-induced cell death. *Photochem Photobiol* **68**: 101–109

Sheline GE, Wara WM and Smith V (1980) Therapeutic irradiation and brain injury. *Int J Rad Oncol Biol Phys* **6**: 1215–1228

Schmidt MH, Bajic DM, Reichert KW, Martin TS, Meyer GA and Whelan HT (1996). Light-emitting diodes as a light source for intraoperative photodynamic therapy. *Neurosurg* **38**: 552–556

Schultheiss TE, Kun LE, Ang KK and Stephens LC (1995) Radiation response of the central nervous system. *Int J Rad Oncol Biol Phys* **31**: 1093–1112

Tyrrell R (1999) Redox regulation and oxidant activation of heme oxygenase-1. *Free Rad Res* **31**: 335–340

 *© 2000 Cancer Research Campaign*

White E (1996) Pathway of regulation of apoptosis: Overview of apoptosis. *Calbiochem Novabiochem Int* **1**: 8–15

Wong CS, Van Dyk J, Milosevic M and Laperriere NJ (1994) Radiation myelopathy following single courses of radiotherapy and retreatment. *Int J Rad Oncol Biol Phys* **30**: 575–581

Yoshida Y, Dereski MO, Garcia JH, Hetzel FW and Chopp M (1992a). Neuronal injury after photoactivation of Photofrin II. *Am J Path* **141**: 989–997

Yoshida Y, Dereski MO, Garcia JH, Hetzel FW and Chopp M (1992b) Photoactivated Photofrin II: Astrocytic swelling precedes endothelial injury in rat brain. *J Neuropath Exp Neurol* **51**: 91–100

Zaidi SIA, Oleinick NLL, Zaim MT and Mukthar H (1993) Apoptosis during photodynamic therapy-induced ablation of RIF-1 tumors in C3H mice: Electron microscopic, histopathologic and biochemical evidence. *Photochem Photobiol* **58**: 771–776

*© 2000 Cancer Research Campaign*
